# Immunoepigenetics Combination Therapies: An Overview of the Role of HDACs in Cancer Immunotherapy

**DOI:** 10.3390/ijms20092241

**Published:** 2019-05-07

**Authors:** Debarati Banik, Sara Moufarrij, Alejandro Villagra

**Affiliations:** Department of Biochemistry and Molecular Medicine, School of Medicine and Health Sciences, The George Washington University, 800 22nd St NW, Suite 8880, Washington, DC 20052, USA; dbanik@email.gwu.edu (D.B.); smm236@email.gwu.edu (S.M.)

**Keywords:** histone deacetylases, immunotherapy, cancer, combination therapies

## Abstract

Long-standing efforts to identify the multifaceted roles of histone deacetylase inhibitors (HDACis) have positioned these agents as promising drug candidates in combatting cancer, autoimmune, neurodegenerative, and infectious diseases. The same has also encouraged the evaluation of multiple HDACi candidates in preclinical studies in cancer and other diseases as well as the FDA-approval towards clinical use for specific agents. In this review, we have discussed how the efficacy of immunotherapy can be leveraged by combining it with HDACis. We have also included a brief overview of the classification of HDACis as well as their various roles in physiological and pathophysiological scenarios to target key cellular processes promoting the initiation, establishment, and progression of cancer. Given the critical role of the tumor microenvironment (TME) towards the outcome of anticancer therapies, we have also discussed the effect of HDACis on different components of the TME. We then have gradually progressed into examples of specific pan-HDACis, class I HDACi, and selective HDACis that either have been incorporated into clinical trials or show promising preclinical effects for future consideration. Finally, we have included examples of ongoing trials for each of the above categories of HDACis as standalone agents or in combination with immunotherapeutic approaches.

## 1. Introduction

Immunotherapy has become standard of care for the treatment of various cancers. Breakthroughs in immunomodulators, such as the use of anti-CTLA4 and anti-PDL-1, have placed more emphasis on targeted immunotherapy as a potential replacement or addition to the standard chemotherapy and targeted therapies. However, immunotherapy can cause toxic and potentially fatal adverse effects [1,2]. Furthermore, not all patients respond well to immunotherapy, which could be due to the varied expression of immune checkpoint molecules in the tumor microenvironment (TME) as well as to the development of resistance towards certain therapeutic drugs. For example, analyzing tumors from patients that did not respond to ipilimumab (anti-CTLA4), various mutations along the interferon-gamma pathway genes (including interferon gamma receptor 1 and 2) were discovered to play a role in promoting tumor cell escape from T cells [3]. Additionally, it has been found that certain genes relating to mesenchymal transformation, wound healing and stemness are expressed in tumors that do not respond to anti- PD-1, an example of this is pancreatic cancer [4]. Epigenetics has a role to play in cancers that are resistant to immunotherapy, whether as conjunctive therapy or as substituting agents. Epigenetic changes are quite common in the TME, leading to various changes in gene expression causing tumor escape. Supplementing immunotherapy with epigenetic modulators, such as histone deacetylase inhibitors (HDACis), may provide substantial benefits, which include, but are not limited to, decreased risk for immune-related adverse effects, upregulating the immune response, and altering the drug resistance within the TME. In recent studies, different HDACis have been found to decrease tumor progression further when combined with immune checkpoint blockade (ICB) [5,6,7,8,9,10]. However, due to the variability in the cellular makeup of the TME and expression of immunomodulatory mediators, such as cytokines, MHC complexes, and tumor antigen markers, the treatment outcomes were found to vary drastically. Although the exact molecular mechanisms enhancing the antitumor activity are not fully understood, it has been reported that multiple components of the TME are affected by epigenetic modifiers. In this review, we will discuss multiple immunological consequences of using HDACis as immunomodulatory agents in standalone therapy or in combination with immunotherapy. Emphasis is given in evaluating the importance of different components of the TME and how the information can be leveraged to enhance the efficacy of combination therapy. We have attempted to capture the dynamic nature of the interplay between the epigenetics and immune system, summarized as the term immunoepigenetics, which is context-dependent and has a signature role in determining the disease outcome [11]. In this review, we have briefly explored the interaction of HDACi within the specific compartments of immune cells. Examples of the critical players in TME include T cell subsets [12,13,14,15], NK cells [16], macrophage [10,17,18,19], B cells [20,21], dendritic cells [22,23], and members of specific innate immune cells [24], such as mast cells [23,25] and granulocytes [26,27].

## 2. Classification and Importance of HDACs

DNA is packaged into nucleosomes wrapped around histone octamers. These histones can be modified in different ways to regulate their accessibility to transcription factors. The histone acetylation/deacetylation cycle, carried out by histone acetyltransferases (HATs) and histone deacetylases (HDACs), is a critical component in this process. HATs promote acetylation of the positively charged N-terminal lysine residues on the histones, which prevent tight binding to the negatively charged DNA strand, thereby promoting an open chromatin configuration and permitting gene transcription [28,29]. On the other hand, deacetylation, mediated by HDACs, favors tight binding of DNA to the histone proteins, thus favoring compact chromatin, which is associated with prevention of gene transcription [29]. 

HDACs are divided into various classes based on their homology to yeast HDACs [28]: Class I is made up of HDACs 1, 2, 3, and 8, which are located within the nucleus. Class II comprises HDACs 4, 5, 6, 7, 9, and 10, which are located in both the nucleus and the cytoplasm and is further subdivided into Class IIa (HDACs 4, 5, 7, and 9) and class IIb (HDACs 6, 10). Class IV is represented by HDAC11. Class III HDACs are composed of seven sirtuins SIRT1-7 and are NAD+ dependent protein deacetylases localized in the nucleus (SIRT 1, 6, and 7), mitochondria (SIRT 3, 4, and 5) and cytoplasm (SIRT 2) [30]. Classes I, II, and IV contain zinc molecule an essential cofactor, while Class III requires NAD+ as a cofactor. HDACs, affect gene expression by acting on various targets including histone proteins as well as transcription factors and regulators, signal transduction mediators, DNA repair enzymes, chaperone proteins as well as nuclear import regulators. 

## 3. HDACis at a Glance

The balance between HAT/HDAC expression and activities is critical in maintaining cellular function. For example, changes in HAT/HDAC activity can affect specific signaling pathways, such as proteasomal degradation, and alter DNA methylation status, which, in turn, affects gene expression [31]. Upregulation of specific subclasses of HDACs has been seen in different cancers. For example, HDAC1 is highly expressed in prostate, gastric, colon, breast, lung, and esophageal cancers; HDAC3 is overexpressed in colon and breast cancers; HDAC6 is upregulated in mammary tumors; HDAC8 is overexpressed in neuroblastoma cells; HDAC11 is found mainly in rhabdomyosarcoma; and HDAC2 has been reported to be elevated in gastric, cervical, and colorectal malignancies [32,33]. The collective data suggest that HDACs play significant roles in gene silencing, which can facilitate malignant cell growth and survival by preventing the expression of cell cycle checkpoints, downregulating the immune response and reducing apoptosis. HDACs also play significant roles in various molecular pathways, including development and differentiation. For example, HDAC 2 knockout mice are born with detrimental cardiac defects that cause their demise within 24 hours [34]. Similarly, HDAC 3, 5, and 9 knockout mice models are characterized by hypertrophic cardiac defects and fibrosis [30]. Furthermore, HDAC8 knockout mice, while able to sustain life, are born with severe craniofacial defects [35]. This discrepancy demonstrates a complex role held by HDACs in both oncogenesis and normal cellular or tissue development.

HDACis can be classified into five distinct classes based on their molecular structures: hydroxamic acids, short chain fatty acids, benzamides, and cyclic tetrapeptides, and sirtuin inhibitors [31]. HDACis may have different activities on transformed cells compared to normal cells, which further testify to the fact that cancer cells are at greater epigenetic vulnerability when compared to normal cells, suggesting that HDAC machinery might be crucial for survival and growth of cancer cells as opposed to normal cells. HDACis can change gene expression by altering transcription of various proteins via the acetylation of histones, transcription factors and a vast array of proteins. Although some HDAC are overexpressed in cancerous tissue, there is no established causal relationship of HDAC overexpression to oncogenic changes. However, knockdown models of HDACs have shown to promote antitumor effects via cell cycle arrest, inhibition of angiogenesis, induction of apoptosis and autophagy, as well as modulation of immune response [20].

Pan-HDACi, such as vorinostat, panobinostat, and valproic acid, are designed to inhibit the HDAC I, II, and IV. Class-specific HDACis, such as MS275 (a class I HDAC inhibitor), MC1568 (a class III HDACi), sirtinol (a class II HDACi), and Nexturastat A (HDAC6i), among many others, have been used in more recent work attempting to decrease the adverse effects related to pan-HDAC inhibition. In all three cases, a vast spectrum of biological effects is observed due to the unique chemical structure and mechanistic profiles of each of the inhibitors [36]. 

## 4. HDACis as Therapeutic Agents in Immune and Nonimmune Diseases

HDACis have been found to modulate different components of the immune system and have been postulated as potential therapeutic agents for various autoimmune and inflammatory diseases [37]. In a mouse model of systemic lupus erythematosus (SLE), it was found that treatment with pan-HDACi trichostatin A (TSA) led to a decrease in mRNA expression of various inflammatory cytokines such as IL-6, IL-2, IL-10, and IFNγ [38]. Due to their anti-inflammatory properties, TSA and suberoylanilide hydroxamic acid (SAHA) (also known as vorinostat) were used to assess the reduction in cytokine-mediated cellular destruction of pancreatic beta cells in a Type I Diabetes model [39,40]. However, it is important to note that while HDACis have been shown to have anti-inflammatory properties, this characteristic is dose-dependent. A similar dichotomy is seen when evaluating the role of HDACis in multiple sclerosis. TSA was also shown to act as an anti-inflammatory mediator in a murine model of multiple sclerosis (MS), decreasing the production of cytokines and thus leading to protection of the myelin [38]. However, another similar HDACi—sodium butyrate—was found to increase metalloproteinase-9, a pro-inflammatory enzyme in MS [38]. HDACis have also been studied in the setting of rheumatoid arthritis and inflammatory bowel disease as regulators of the immune response, modifying monocyte and macrophage response to disease [41,42,43]. Class I HDACis were found to decrease the synthesis of inflammatory cytokines in the synovial joint, an accurate model for rheumatoid arthritis [41]. Furthermore, HDACis were found to decrease TNFα alpha and IFNβ in models of inflammatory bowel disease both in vitro and in vivo [44]. 

HDACis also contain non-immune related properties beneficial to the improvement of cognition and memory, mainly via reversing transcriptional silencing at particular disease loci. For example, HDAC6 inhibitor SW-100 was found to enhance memory in a murine model of Fragile X Syndrome by rescuing the expression of fragile X mental retardation protein (FMRP), which is silenced in this disease [45]. Another mechanism by which HDACis can reverse neurodegenerative diseases is through axonal transport. It was found that HDACis can recover mitochondrial axonal transport in hippocampal neurons by decreasing Aß deposition and thus decreasing inflammation in Alzheimer models in vitro and in vivo [46]. Thus, as can be noted, their wide array of mechanistic modulation allows them to act as potential therapeutic alternatives for various chronic diseases.

## 5. HDACis as Anticancer Agents

Due to the antitumor activity of HDACis, these compounds have been extensively studied as alternatives to current anticancer agents. Vorinostat is the first pan-HDACi to be approved for the treatment of relapsed and refractory cutaneous T cell lymphoma, and romidepsin, a pan-HDACi, is approved for the treatment of peripheral T cell lymphoma [47]. Similarly, belinostat is part of the therapeutic regimen for peripheral T cell lymphoma while panobinostat has been studied in the setting of multiple myeloma (MM) [31]. In many cases, the nature of cancer development is associated with abnormal acetylation on histones conferring antitumor properties to the HDACis and making them appealing therapeutic candidates. It has been found that global loss of acetylation of histone H3 lysine and histone H4 is associated with poor prognosis in breast, prostate, and lung cancer [48]. HDACis have demonstrated synergistic or cumulative anticancer effects when combined with various antitumor agents, such as chemotherapy and radiation. An example of this is the use of HDACi with topoisomerase II inhibitors. Prior treatment with HDACi created an environment favoring chromatin decondensation, allowing for optimal topoisomerase II inhibitor action to cleave DNA, resulting in synergy in the combined drug cocktail. Similar findings have been demonstrated with 5-fluorouracil, gemcitabine, docetaxel, and cisplatin [49]. 

Listed below are some of the cellular/physiological pathways that involve HDAC-mediated regulation, making them critical in cancer pathology.

### 5.1. Cell Cycle Arrest

HDACis have been found to induce cell cycle arrest by upregulating the expression of cyclin-dependent kinase inhibitor (CDK inhibitor) families such as p21, which in turn induces G1 arrest [50]. P21 blocks the formation of dimers from cyclins and cyclin-dependent kinases, thus inducing cell cycle arrest and inhibiting cell differentiation [51]. Furthermore, HDACis inhibit the deacetylation of p53 protein, another CDK inhibitor, further increasing its half-life and improving its interaction with p21 [52]. This epigenetic modification has essential ramifications in oncogenesis as dysregulated cell growth is known to be a significant factor in tumor malignancy. Recent studies have reported that the deacetylation of p53 [53] and PTEN [54] proteins by HDAC6 decrease their activity and target them to proteasome degradation. These two studies hypothesize that inhibiting HDAC6 sustains the tumor suppressor activity of p53 and PTEN proteins and therefore, HDAC6i could benefit as antitumor agents only in tumors expressing p53 and PTEN. 

### 5.2. Angiogenesis

HDACis have been shown to downregulate angiogenesis through various mechanisms. HDACis can hyperacetylate hypoxia-inducible factor HIF-1α, a proangiogenic transcription factor, causing it to degrade [28,31]. They can also act on the upregulation of anti-angiogenic proteins such as thrombospondin-1 and activin A. Valproic acid (VPA), a pan-HDACi, can increase the expression of both genes by downregulating proangiogenic factors such as fibroblast growth factor [55]. 

### 5.3. Apoptosis

HDACis play a role in activating both the intrinsic and extrinsic apoptotic pathways, which are biological processes that allow for the elimination of damaged cells. HDACis can increase the expression of death receptors and their ligands in malignant cells but not in normal cells [56]. Furthermore, HDACis can promote the intrinsic apoptotic pathway by increasing the release of cytochrome c from the mitochondrial membrane, leading to the activation of caspase-9 [56].

### 5.4. Autophagy

It has been found that HATs and HDACs mediate the acetylation of various autophagy-related proteins [31]. HDACis can upregulate the production of reactive oxygen species, which in turn can induce cell death in apoptosis-resistant cells. An example of this can be seen with vorinostat, a pan-HDACis, and its effect on upregulating ROS and causing cell death in hepatocellular carcinoma and glioblastoma cells [31]. This biological mechanism allows for the stabilization of dysregulated cells when mechanisms of apoptosis cannot prevent unstructured growth.

### 5.5. Modulating Immune Response

HDACis can alter the expression of molecules that upregulate the immune system, such as MHC and costimulatory molecules, which in turn upregulates antigen presentation, thus, in turn, activating T cells. Specifically, it has been found that HDAC6i stimulates naïve T cell functionality and class II HDACis target Tregs. Class I HDACis target adaptive immunity by promoting the functionality of natural killer and CD8 cells [36]. Additionally, HDAC6i has been recently reported to improve ICB in melanoma [10]. The following section will provide further details on this aspect from the standpoint of cancer.

A summarized view of various pan and selective HDACi, their specificity and current standing in the clinical trials are listed in Table 1. "+" indicates inhibitory selectivity. Increased inhibition is marked by a higher "+" designation in an empiric fashion.

## 6. HDACis as Immunomodulatory Agents

The following section will give an overview of the relevance of HDAC/HAT pathways in the regulation of soluble and cellular components within the immune system. The outcomes are particularly important since they determine the composition and behavior of the TME. 

### 6.1. Cytokines

Cytokines occupy a crucial junction between the innate and adaptive immune system. Not only do they initiate immune reactions, but also regulate the ongoing immune processes. Subpopulations of the adaptive immune system, such as effector and helper T cells as well as the antigen-presenting cells, are particularly influenced by the effects of cytokines. Much of the relationship between HDACis and cytokines in inflammation and immune system has been worked out in autoimmunity or infections settings. For example, T cell-polarizing cytokines that play critical roles during intra- and extracellular pathogenesis, e.g., IL12 and IL23, were found to be regulated by TSA, with the former acting on IRF1 to decrease its recruitment to the IL-12 P40 promoter and decrease IL-12p40 mRNA expression [57]. Several other cytokines that sit at the crucial junctions of cancer and autoimmunity are susceptible to HDACi regulations as well, for example, IL6, IL8, MIP1, TNFα, IFNγ, and IFNβ [58]. In conjunction with TLR stimulation, HDACi has been shown to inhibit several pro- or anti-inflammatory cytokines, such as COX2, IL12, IL23, and IFNγ [59,60]. Additionally, HDACis have been shown to increase the expression of IDO1, an immunoregulatory enzyme that decreases the expression of dendritic cells via acetylation of histones (40). HDACis also have been explored as anti-inflammatory modulators to revert autoimmune or inflammation-based conditions. The pan HDACi SAHA demonstrated significant effect even on the circulating cytokines, such that a single oral administration of SAHA to mice dose-dependently reduced circulating TNFα, IL-1β, IL-6, and IFNγ induced by lipopolysaccharide (LPS) as well as reduced the release of TNFα, IL-1β, IL-12, and IFNγ from the LPS-stimulated human PBMCs in vitro [61]. Finally, selective HDAC6i has been shown to modulate the expression of IL-10 and other cytokines in macrophages and dendritic cells [62]. 

### 6.2. Antigen-Presenting Components

Antigen-presenting cells (APCs) play a central role to modulate immune reaction by processing the antigens, forming complexes with either MHC class I or class II and presenting the bound peptides to the CD8+ or CD4+ T cells, respectively. In APCs, the proteasomes process the peptides that are transported by the ATP-dependent heterodimeric transporter associated with antigen processing transporters (TAP-1 and TAP-2) machinery into the endoplasmic reticulum (ER). In the ER, the antigen fragments are loaded onto MHC class I. The stabilized peptides/MHC I complexes are then translocated to the cell surface to interact with the CD8+ cytotoxic T-lymphocytes (CTLs). Ideally, it is only MHC molecules loaded with peptides derived from tumor-associated antigens (TAA) that should be able to activate CTLs. These antigens are expressed by malignant cells or peptides derived from foreign pathogens, and therefore, the cellular immunity is designed to be a tool for elimination of malignant, foreign, and infected cells [63,64]. By losing the antigen-presenting machinery components, malignant cells tend to evade the detection from the adaptive immune system. MHC class I abnormalities are found in solid tumors of distinct origin as well as in hematopoietic diseases. Specific examples of such abnormalities that can occur at the epigenetic, transcriptional, and post-transcriptional levels include [1] structural alterations such as total, haplotype, and allelic loss of the MHC class I heavy chain; 2) deletions and point mutations, e.g., in beta2-microglobulin and TAP1; and [3] dysregulation of various components of the MHC class I antigen processing machinery (APM) [65]. MHC class I antigens and the various APM components are constitutively expressed in all normal tissues, except embryonic cells, testes, and ovaries. Under physiological conditions, the expression of the MHC class I APM components are regulated in a tissue-specific manner, often dependent on the differentiation and cell cycle stages [65]. 

The components mainly involved in antigen presentation, such as MHC class I HC, β2-m, tpn, and the TAP1/TAP2 heterodimer, are predominant in mature DC compared to immature DC [66]. Induction of differentiation with retinoic acid, valproic acid, and TSA could modify the expression pattern of some of these molecules [65,67,68]. Different cancer types employ different modes of subversion for the APM machinery, which includes structural alterations and dysregulation due to epigenetic control, and transcriptional and posttranscriptional modulation. Also, alterations include mutations in tpn and/or LMP subunits in neuroblastoma and melanoma, e.g., point mutations or base pair deletions [65]. Genetically, the majority of the APM components are located on the same chromosome close to the MHC locus, and are thereby regulated by similar promoters, indicating an evolutionary mechanism of induction in a coordinated manner. Master regulators, such as IFNγ is an inducer of the whole cluster of genes [69,70]. Therefore, loss of MHC class I towards IFNγ inducibility may result from the alterations in the IFN pathway itself [71]. Various tumor types, such as esophageal squamous cell carcinoma, colon carcinoma, RCC, and melanoma cell lines, demonstrate epigenetic changes such as methylation and histone deacetylation of APM components. Some of these modulations could be reverted by treating with histone deacetylase inhibitors of DNMT inhibitors [72]. 

For example, using TAP-deficient metastatic cell lines, it was shown that TSA treatment enhances expression of multiple APM components and surface expression of MHC I molecules, which in turn, enhances the susceptibility of the tumors towards CTL-mediated killing. One such mechanism previously identified was the enhanced recruitment of RNA Pol II to the promoter of TAP1 [64]. Pan-HDACis has been shown to increase various molecules in the APM pathway, such as TAP-2, tapasin, calnexin, calreticulin, ERp57, LMP2, and LMP7 [73,74]. 

Dendritic cells (DCs), the primary mediator for antigen presentation, act as a bridge between adaptive and innate immune systems. Circulating in peripheral tissue in their immature state, DCs continually capture antigens, converting into APCs only when they receive the maturation signal from the damaged or infected tissues. Migrating to the secondary lymphoid organs, the APCs then interact with naïve T cells and supply them with signal 1 (MHC-peptide complexes), signal 2 (costimulatory molecules), and signal 3 (cytokines for directing the polarization of T cells into subsets such as Th1, Th2, Treg, and Th17). DCs are the source of at least three essential costimulatory signals, in the absence of which, the T cells enter the state of tolerance or anergy. These are CD40 (that binds to CD40L on T cells) and CD80 and CD86 (that bind to CD28 on T cells). Several pan-HDACis have shown a suppressive effect on the expression of both signal 2 and signal 3 by DCs. For example, MS-275 and VPA reduce expression of costimulatory molecules, cytokine production, and signaling pathways through NFκB, IFR8, and IRF3 [75]. Other HDACi such as SAHA, LBH589, apicidin, ITF2357, and TSA are also reported to suppress the expression of costimulatory and adhesion molecules in DC, both in vivo and in vitro [57,76,77]. Cytokines secreted by DCs are responsible for either priming (e.g., IL-1b, IL-6, IL-15, and TNFα) or polarization (e.g., IL-12, IL-18, and IL-7) of T cells. Pan-HDACis also play profound roles in suppressing these signals. For example, MS-275 was reported to reduce TNFα, IL-6, and IL-12, as well as IL10 in response to poly I-C [75]. Another pan-HDACi LBH589 blocks the production of IL-6, IL-12p70, IL-23, TNF and IL-10, by the TLR3- and TLR4-activated DCs [60]. Another study suggests that HDACi may regulate DC function by acetylation of STAT3, which in turn may enhance indoleamine 2,3-dioxygenase 1 (IDO1) production by DCs [78]. IDO1 is an immunomodulatory enzyme produced by some regulatory cells, which is responsible for the catabolism of tryptophan that is essential for T cell activation [79,80]. All these factors strongly suggest that HDACis mediated effect on APCs may regulate the fate of both innate and adaptive immune system as well as the prevailing inflammatory milieu critical in cancer.

### 6.3. Subsets of T Cells

#### 6.3.1. Treg

Thymic production of murine FOXP3^+^ Tregs as well as the peripheral conversion of T cells into Tregs can be promoted by pan-HDACis (e.g., TSA or SAHA), and the Treg suppressive function can be stimulated both in vitro and in vivo [81]. After TSA treatment in mice, FOXP3+ CD4+ T cells increase significantly within the thymus and secondary lymph nodes. Although the CD25 expression level remains comparable, the expression of the FOXP3 gene is enhanced after TSA treatment, with a concomitant increase in the suppressive function in the CD25+ FOXP3+ subset of CD4 T cells. Acetylation of the FOXP3 protein at specific lysine residues in the forkhead domain promotes the DNA binding ability of FOXP3 contributing to the suppressive activity of Tregs. In the context of T cell suppressive pathways, HDACis upregulate the expression of CTLA4 on human Tregs, enhancing their suppressive activity on T effector cells [12]. Some of the well-known pan-HDACis also enhance the Treg population. For example, SAHA and MS-275 may convert anti-CD3/anti-CD28-stimulated human CD4(+)CD25(−) T cells into FOXP3+ suppressive Tregs [82]. 

#### 6.3.2. Th0, Th1, and Th2 Subsets and Their Interconversion

Epigenetic modulations through pan-HDACis are powerful mediators to determine the Th1 vs. Th2 balance. For example, pan-HDACis, such as LAQ824, alter Toll-like receptor 4 (TLR4)-dependent activation and function of macrophages and dendritic cells (DCs), resulting in the activation of a distinct sets of genes [83]. Specifically, this small molecule inhibits T helper 1 (Th1) effector but not Th2 effector cell activation and migration. Moreover, it inhibits the chemotaxis of macrophage- and DC-mediated monocytes, but not neutrophils. These findings indicate a high specificity of HDAC inhibition in modulating innate and adaptive immune responses. LAQ824 blocked IFN-γ secretion by Th1 cells in the presence of LPS-stimulated DCs in a dose-dependent manner. However, when Th2 effector cells were incubated with stimulated DCs, LAQ824 failed to modulate IL-4 secretion by the T cells. Genome-wide gene expression analysis of LAQ824-treated APCs also revealed a small set of genes to be differentially regulated in the presence of pan HDACis. The rapidity of onset of the cell cycle after stimulation of naïve T cells with IFNγ or IL4 suggested HDAC-mediated epigenetic control over the process [84,85]. Pan-HDACi TSA is shown to promote acetylation of IFNγ promoter within the murine Th0 cells [85]. Rapid downregulation of markers such as CD28 and CD62L are shown to be mediated by pan-HDACis, supporting the hypothesis that reversion of the acetylation status offers a rapid mode for T cell polarization during the early activation phase of genes [12,86]. 

Furthermore, during the IL12/IL4-driven polarization of T cells into Th1/Th2 subtype, specific transcription factors, such as T-bet (in Th1 cells) and GATA-3 (in Th2 cells) become particularly crucial, in addition to other factors such as STAT4 and STAT6. Stable lineage commitment is primarily achieved by sequential acetylation events, such as acetylation of IFNγ promoter before the acetylation of T-bet. The initial acetylation events in this sequence ensure specific modifications of cytokine and STAT pathways, heavily dependent on the HAT and HDAC enzymes for achieving a pure Th1 or Th2 state [87,88]. 

#### 6.3.3. Th17

The specific subtype of T cells that is characterized by secretion of IL17A and 17F, also termed Th17 lineage cells, is particularly dependent on the expression and function of the transcription factor RORyT. It is the master regulator that determines the differentiation of CD4+ towards Th17. In a recent work on the Th17 lineage, a distinct epigenetic regulation was identified for the expression of RORγT from RORC gene [89]. Using naïve CD4+ T cells under Th17 differentiation conditions, it was found that two pan-HDACis (butyrate and apicidine) were able to cause a significant decrease in the RORγT expression, accompanied by acetylation of the H4 molecules proximal to the RORγT promoter. This observation was aligned with the earlier finding in a murine model with HDACi ITF2357, shown to inhibit the IL6/IL6R pathway, which shifts the differentiation of CD4+ T helper cells into Tregs instead of Th17 [90,91]. Another mode of HDAC-mediated regulation is the post-translational reciprocal regulation of RORγT by the histone acetyltransferase (HAT) activity of p300 and HDAC1. P300 was described to interact with and acetylate RORγt at its K81 residue whereas HDAC1 removes it to continue the cycle of transcriptional regulation of IL17 [92].

### 6.4. Natural Killer (NK) Cells

NK cells express a broad range of activating and inhibitory receptors which help them recognize and engage with targets for subsequent cytotoxic and lymphokine release functions [93]. VPA was shown to upregulate both protein and mRNA expression of major histocompatibility complex class I-related chain (MIC) A/B and UL16-binding protein (ULBP) 2, without any significant effect on the expression of ULBP1, ULBP3, and ULBP4 or induction of other NK cell ligands such as NKp30-L, NKp44-L, and NKp46-L [94]. In another study, both VPA and SAHA were shown to inhibit IL2-activated NK cell function by decreasing the expression and functions of NKp46 and NKp30, as well as by impairing the exocytosis of granules [95]. The NK receptor NKG2D is also found to be responsive to HDAC inhibition. For example, the transcription of NKG2D can be enhanced by H3 and H4-acetylation by entinostat [96]. HDAC3 plays a critical role in NKG2D transcription through its partnership with STAT3 and subsequent phosphorylation of STAT3 at the Tyr 705. VPA can inhibit HDAC3, thereby eliminating the phosphorylation of STAT3 and regulating NKG2D expression [97]. VPA is also shown to downregulate NK cell-mediated antitumor cytotoxicity by inducing histone K9 hypermethylation and DNA methylation in the promoter of NKG2D gene [98].

## 7. Advantages of Immunotherapeutic Combinations using HDACis

Historically, the progression of cancer has been treated with anticancer agents with cytotoxic properties. Examples of such compounds include taxol (which influences the microtubules of the cells [99], anthracyclines (which inhibit the function of Topoisomerase II [100], epothilones (which perturb cell cycle and cell division [101]), halichondrin analogs (which inhibit micro-tubulin polymerization [102]), and platinum compounds (which produce DNA damaging effects in cells [103]). However, the therapeutic index (a ratio that compares the blood concentration at which a drug becomes toxic and the concentration at which the drug is effective of such agents) tends to be narrow for these targeted therapies [104]. Moreover, such systemic therapies (e.g., chemotherapy and hormonal therapy) are mostly applied intravenously or orally, without discriminating between malignant and normal tissues [105]. As a result, naturally or rapidly renewing tissues, such as the bone marrow and gastrointestinal tract, are particularly left susceptible to the cytotoxic agents causing recurrent cases of secondary hematologic and solid tumors [106]. On the other hand, targeted therapies also assist in acquiring multidrug resistance, due to enhanced genomic instability and selection of resistant clonal populations [104,105]. As a recent alternative, immunotherapy has emerged as a powerful modality to rejuvenate the immune system and mount active immune-responses against tumor leading to clinically observable benefits. However, several barriers prevent immunotherapy from reaching its maximum efficacy. Such factors include, downregulation of MHC molecules and tumor antigens, development of a suppressive microenvironment by upregulation of anti-inflammatory cytokines and accumulation of immuno-suppressive cells (such as cancer-associated fibroblasts, regulatory T cell subset among the CD4+ T cells, myeloid-derived suppressor cells, tumor-associated macrophages, and tumor-associated neutrophils), and tumor-induced immune senescence that prevents the antitumor activities of the cytotoxic cells [107].

A classical study that initiated the interest in the combination of HDACis with immunotherapy was a dual application of two epigenetic modifiers, e.g., entinostat and azacytidine. The first study with this combination remained unsuccessful in producing a significant antitumor response in a cohort of lung cancer patients [108]. A subset of these patients, however, went on to participate in an anti-PD1 based trial with nivolumab. Interestingly, five out of the six patients showed a progression-free survival 6 months post-treatment, an unexpected outcome in the non-small cell lung carcinoma disease [109]. Encouraged by this observation, many cancer types have been explored so far both in preclinical and clinical settings, such as melanoma, prostate, colon cancers, using either pan or selective HDAC inhibitors [108]. A specific preclinical example of this approach is found in a recent study: where significant improvement of antitumor immune responses when combing anti-PD-1 and ultra-selective HDAC6i was identified [10]. According to this study, tumor growth along with tumor infiltrated cells and cytokine milieu were modified as a result of the combination treatment, making it more susceptible to immunotherapy. This recent preclinical study supporting the prior entinostat/azacytidine example, elaborates the need to use epigenetics in the context of priming the microenvironment for a better success with subsequent application of immunotherapy.

Several innovative modes of immunotherapy are being tested against cancer: cytokine-mediated [110], adoptive cell-mediated [111], receptor agonist antibody mediated [112,113], cellular vaccine-mediated [114,115], and immune checkpoint mediated [116,117,118], just to name a few. HDACis have shown to enhance the efficacy of some of these modalities in many unique ways:

1. *Enhancement of the expression of cancer antigens:* The expression of tumor antigens and the surface expression of MHC and costimulatory genes are critical determinants in T cell activation. Epigenetic repression of these molecules in tumor cells provides a mechanism for tumor escape, in which HDAC enzymes may play instrumental roles [119,120,121]. Employing HDACis have proven to enhance tumor antigens (e.g., MAGE [122]) and costimulatory molecules (e.g., CD86 and ICAM1 [123]).
2. *Epigenetic modulation of the immunosuppressive cell population:* Regulating inflammation is one of the primary functions attributed to pan-HDACis. Upregulation of the FoxP3 gene and fostering Treg generation and function serve as a novel mechanism by which histone deacetylase inhibitors regulate the inflammation and immune response [82]. On the other hand, using myeloid-derived suppressor cell (MDSC)-rich tumors, the importance of HDACis treatment has been shown to decrease MDSC accumulation in the spleen, blood and tumor bed, and conversely, increasing the proportion of T cells [124].
3. *Modulation of specific suppressive pathways:* Some of the prominent immunosuppressive pathways that dampen T cell functions include the induction of the metabolic enzyme indoleamine-2,3-dioxygenase 1 (IDO1). Other mechanisms of immune-resistance include innate oncologic molecular pathways or their dysregulation. Such examples include β-catenin [125], STAT3 [126], NF-κB [127], PTEN [128], and AXL tyrosine kinase [129]. Some of the inhibitory interactions include programmed cell death 1 (PD-1) with its ligand PD-L1, and engagement of additional inhibitory receptors such as T cell immunoglobulin and mucin domain-containing-3 (HAVCR2 or TIM3) [129,130]. In many cases, the effect of broad spectrum HDACi acts in favor of the immunosuppressive pathways. For example, vorinostat was found to reduce pro-inflammatory cytokines through the induction of indoleamine-2,3-dioxygenase 1 (IDO1) in a STAT-3-dependent manner [59] and increased regulatory T cells (Tregs) [81].
4. *Induction of specific chemokine expression on T cells:* Expression of chemokines ensures T cell motility inside the tumor microenvironment, and pan-HDACis have been described to enhance the expression of specific chemokines. Specific examples include Ccl5, Cxcl9, and Cxcl10 induction by romidepsin and vorinostat [14]. Vorinostat has also been shown to induce the IL-8/CXCL8 expression in ovarian cancer cells, which is dependent on IκB kinase (IKK) activity and is associated with gene-specific recruitment of IKKβ and IKK-dependent recruitment of p65 NFκB to the IL-8/CXCL8 promoter [131]. The potential advantages of using immunotherapy-based pharmacological combinations and a few relevant examples are summarized in Table 2. The following sections will specifically address the HDACi classes particularly explored with immunotherapeutic combinations under preclinical and clinical settings in cancer pathogenesis.

## 8. Pan-HDACis & Their Involvement/Success in Immunotherapy Combination Modality

In 2006, SAHA (vorinostat, zolinza™, Merck & Co, Inc., city, state, USA) was approved by the FDA as the first HDACi for the treatment of cutaneous T cell lymphoma (CTCL) [106]. In 2004, belinostat (PXD101, BELEODAQ™, Spectrum Pharmaceuticals, Inc.) was approved by the FDA to use against peripheral T cell lymphoma (PTCL) [132]. Panobinostat (Farydak, Novartis Pharmaceuticals) was approved in 2015 for the treatment of multiple myeloma [133]. The following section gives some examples of the pan-HDACis molecules that have found significant use in combination with different immunotherapeutic modalities.

### 8.1. Valproic Acid

Valproic acid has been shown to serve as a successful therapeutic agent when used in combination with immune modulators and other HDACi. For example, pan-HDACis AR42 and sodium valproate were shown to alter the immunogenicity of melanoma cells and treatment efficacy [134]. The HDACis were able to reduce the expression of PD-L1 and PD-L2 rapidly and enhanced the expression of MHCA on the melanoma cells. The immunogenic protein HMGB1 was also released into the extracellular environment. Using a murine melanoma model (B16), a pretreatment with either AR42 or sodium valproate was shown to enhance the efficacies of both anti-PD1 and anti-CTLA4 antibodies. Moreover, the antitumor efficacy of a multi-kinase inhibitor pazopanib was also enhanced by sodium valproate. HDACis in combination with anti-PD-1 also enhanced the levels of CCL2, CCL5, CXCL9, and CXCL2, which correlated with increased activated T cell, M1 macrophage, neutrophil and NK cell infiltration [134].

VPA combinations with different chemotherapeutic agents have succeeded in several animal studies. Such as, with capecitabine [135] to treat breast cancer, with cisplatin and cetuximab in recurrent and/or metastatic squamous cell carcinoma of Head and Neck cancer [136], with DNA methyltransferase (DNMT1) inhibitor azacytidine [137] for myelodysplastic syndrome. However, combining VPA with chemo-immune agents such as dacarbazine and interferon-α in a phase I–II study did not produce a better outcome than the standard therapy in patients with advanced melanoma [PMID: 19127265]. 

### 8.2. Panobinostat (LBH589)

According to the previous work, HDAC inhibition by panobinostat induced prolonged PD-L1 expression in both human and mouse melanoma cell lines. Patient melanoma samples showed elevated expression of PD-L1 and PD-L2 in a dose-dependent manner. Due to the inhibition of HDAC, a more relaxed chromatin state at the promoter regions of PD-L1 and PD-L2 was achieved which resulted in increased gene expression. Applying the pan-HDAC inhibitor LBH589 along with PD-1 blockade resulted in reduced tumor burden and improved survival in a murine B16F10 model of established tumors [6]. According to more recent work [138], panobinostat made a significant difference in inducing complete response in multiple myeloma by combining it with anti-CD38 monoclonal antibody (mAb) daratumumab, which was shown to be effective in multiple myeloma. Daratumumab was initially used at first relapse in combination with bortezomib/dexamethasone or lenalidomide/dexamethasone, with the efficacy of inducing complete responses in a notable percentage of patients (19.2% and 43.1%, respectively) [139,140]. According to prior evidence, B cell lymphoma and acute myeloid leukemia showed that panobinostat induces epigenetic changes leading to enhanced expression of CD20 and CD33 and subsequently enhancing the efficacy of mAb immunotherapy [141,142]. In the case of MM, the increase in CD38 expression after panobinostat treatment was specific for myeloma and was not evident in lymphoma cell lines. Also, there was a significant increase in antigen-dependent cellular cytotoxicity in the panobinostat-treated group of patients compared to the untreated. The data indicated that the synergy between panobinostat and daratumumab could be used to enhance response rates and extend the duration of responses in relapsed/refractory myeloma compared with daratumumab alone. 

Due to its prior success in the preclinical setting, panobinostat has been escalated into an ongoing phase 1 clinical trial (NCT02032810) in combination with Ipilimumab for the unresectable melanoma patients, currently being conducted by the H. Lee Moffitt Cancer Center and Research Institute in collaboration with Novartis.

### 8.3. LAQ824

Another hydroxamic acid derivative pan HDACi LAQ824 is shown to enhance the antitumor activity of pmel-1 adoptive transfer immunotherapy. To investigate if LAQ824 could render melanoma cells more susceptible to immunotherapy, LAQ824 was administered together with tumor antigen-specific immunotherapy [143]. According to this in vivo study, treatment with LAQ824 increased antigen presentation by tumor cells and enhanced function of immune cells in pmel-1 model vaccine prime–boost models. Both the pathways resulted in increased antitumor activity as tested in mice with 12-day established B16 tumors. The tumor-bearing host (mean diameter of tumor 5–8 mm) received a lymphodepleting dose of total body irradiation, pmel-1 T cells adoptively transferred intravenously into B16 tumor-bearing mice and gp10025-33 peptide-pulsed dendritic cell vaccination with high-dose IL-2 therapy. It was found that the addition of LAQ824 ameliorated antitumor activity compared with pmel-1 adoptive transfer monotherapy. Combined treatment with LAQ824 and pmel-1 adoptive transfer had a statistically significant prolongation in survival. The administration of HDACi also improved the antitumor activity in a prophylactic vaccine model against B16 melanoma. The treatment resulted in an increase in the intratumoral infiltration & functional activity of adoptively transferred pmel-1 cells. The efficacy of the treatment appeared to be dependent on the antigen-driven expansion of pmel-1 cells via increasing MHC molecules and antigen expression. 

### 8.4. Vorinostat

An increase in the PD-L1 expression was shown to be caused by vorinostat in a preclinical study on TNBC [5]. In this study, mice were injected with vorinostat, a combination of anti-PD-1, and anti-CTLA-4 blocking antibodies. The addition of anti-CTLA-4 to the anti-PD-1 blockade already induced a significant inhibition of tumor growth compared to single-agent treatment, which was further improved by adding vorinostat. Vorinostat alone, the immunotherapy combination (anti-PD-1 + anti-CTLA-4) and the three-drug combination reduced tumor burden by 12.5%, 34%, and 88.5%, respectively. Although repeated treatment with anti-CTLA-4 and anti-PD-1 as single-agent could reduce tumor growth, significant tumor eradication was only observed when the HDACis was combined with the immunotherapy treatment. This synergistic interaction of the three drugs increased survival as well. The combined treatment was well tolerated in the host. While vorinostat decreased the number of FOXP3+ cells, the combination of vorinostat and immunotherapy did not alter that number. However, the triple combination further upregulated the CD8+ T cell population, indicating that the epigenetic modulation and immunotherapy could supplement each other by affecting different properties of the tumor milieu [108].

### 8.5. Belinostat

Belinostat is approved for use in relapsed or refractory peripheral T cell lymphoma (PTCL) [144]. Phosphoproteomic analysis of the belinostat-treated squamous cell carcinoma (SCC) cells showed significant downregulation of MAPK pathway, as well as the induction of apoptosis. Belinostat transcriptionally upregulated the F-box proteins FBXO3 and FBXW10, which downregulate the transcription factor son of sevenless (SOS). SOS facilitates the MAPK pathway contributing to cisplatin resistance in the SCC cells. In the cisplatin-resistant cells with aberrant MAPK activation, combined treatment of cisplatin with belinostat significantly inhibited cisplatin-induced ERK phosphorylation and exhibited strong synergistic cytotoxicity [145]. 

In a continuing phase II trial (NCT01686165) conducted by the University of Arizona and National Cancer Institute (NCI), DLBCL is targeted in combination with belinostat and immune-modulator rituximab, with an additional intervention of Radiation (yttrium Y 90 Ibritumomab tiuxetan: Zevalin). The subsets of disease included in this study are anaplastic large cell lymphoma, recurrent adult diffuse large cell lymphoma, and recurrent mantle cell lymphoma. With a recruitment group of five white participants, the therapy was well tolerated with nausea and vomiting being the most frequent adverse events. Although all patients progressed after receiving therapy, the study did not achieve the required overall response rate (ORR) to proceed to the next stage [146].

## 9. Class I HDACis

Benzamides are the molecular components of Class I HDACis, which include entinostat (MS-275-SND-275), mocetinostat (MGCD0103), romidepsin, RGFP966, and tacedinaline. The molecules mentioned above act at a nuclear level to inhibit HDAC 1, 2, and 3 and are composed of a 2’ aminoanilide moiety that binds to active sites within the HDAC core. MS-275 and MGCD0103 are currently being developed for clinical trials as both single-agent therapy and combination therapy [147]. Mocetinostat, an inhibitor of HDAC1 and 3 has been found to exert antitumor effects in both hematological and solid cancers. In an NSCLC murine model, mocetinostat was shown to upregulate PD-L1, and when used in combination with a murine PD-L1 antibody, significantly decreased tumor burden [7]. Furthermore, mocetinostat upregulated intratumoral CD8+ cytotoxic T cells while decreasing Tregs. In the trial NCT00358982 held on patients with relapsed classical Hodgkin’s lymphoma, it was demonstrated that mocetinostat has promising clinical activity and therapeutic capabilities in patients with classical Hodgkin’s lymphoma, refractory to treatment [148]. 

Entinostat was shown to increase histone H3/H4 acetylation and caspase-3 activation in patients with leukemia [149]. Its primary mechanism of action includes cell death and autophagy and its ability to affect other proteins such as microtubules [150]. 

Furthermore, entinostat was found to reduce tumor burden by inhibiting the TGFβ catenin signaling pathway, a pathway that is overexpressed in colon cancer [151]. Similarly, entinostat has a broad spectrum of activity with a low toxicity profile. Entinostat was found to enhance the antitumor effect of PD-1 inhibition by decreasing tumor growth and improving survival in a mouse model of lung and renal cell carcinoma. Furthermore, entinostat successfully depleted the immunosuppressive function of polymorphonuclear and monocytic MDSC populations, thus shifting from an immunosuppressive to an immune-permissive tumor microenvironment [152]. 

Additionally, entinostat has been evaluated in various models of breast cancer and found to inhibit cell proliferation and promote apoptosis in breast cancer cells, especially if used in addition to antiestrogen management [153]. Romidepsin (FK-228) is approved for the treatment of cutaneous T cell lymphoma as well as peripheral T cell lymphoma [29]. Romidepsin was found to be associated with fatigue, nausea, vomiting, diarrhea, and constipation when assessed in clinical trials; either as a single agent (NCT 00426764) or in combination (NCT03141203). However, when assessed as a single agent or in combination with paclitaxel in the SUM 149 inflammatory breast cancer cell line, it was found to decrease primary tumors and metastatic lesions [154]. RGFP966 is a selective HDAC3 inhibitor involved in targeting DNA replication which was found to decrease tumor growth and promote apoptosis in the setting of refractory CTCL [155]. 

Some of the widely known pan or class-specific inhibitors that have been or currently being used in clinical trials in combination with various immunomodulators are listed in Table 3. 

## 10. Selective HDACis

Selective HDACis can span more than one class but typically target limited HDAC molecules. The move toward targeted HDACi stems mainly from the need to decrease adverse effects which, as aforementioned, are common with pan-HDACis. 

MPT0E028 is an HDAC1, 2, and 6 inhibitor, which has been shown to induce apoptosis in B cell lymphomas [156]. An in vivo model implementing this drug has shown a prolonged survival rate of mice with human B cell lymphoma, and is currently undergoing clinical trials for assessment of its use in patients with advanced solid tumors (NCT02350868). 

Chidamide is a selective HDAC inhibitor targeting HDACs 1,2,3,10. It has been found that chidamide induces apoptosis and cell cycle arrest in various multiple myeloma cell lines [157]. It has also been studied in the setting of relapsed or refractory peripheral T cell lymphoma (PTCL) patients, which found that for patients receiving exclusive therapy with chidamide, the overall response rate was 39.06%, while it was 51.18% when combining chidamide to chemotherapy [158]. Thus, chidamide offers an alternative when used in combination with chemotherapy as it has a low toxicity profile and favorable efficacy in patients with PTCL. 

Ricolinostat, an HDAC6i, has been studied in phase I and phase II clinical trials in the setting of multiple myeloma and lymphoma. Ricolinostat has antilymphoma properties when used as monotherapy as it induces apoptosis in lymphoma cell lines by also increasing cell cycle arrest [159]. Furthermore, it was studied in the setting of multiple myeloma as a phase I/II trial. Patients with refractory or relapsed multiple myeloma, treated with ricolinostat combined with bortezomib and dexamethasone demonstrated minimum adverse effects [160].

As can be noted from the examples above, the selective HDACi’s main anticancer mechanism is cell cycle arrest, which allows them to act as single agents and to potentiate the action of chemotherapy/chemo-immune therapy further.

Some examples of selective HDACis used as a single or combination therapy with various immunomodulator/cell transfer therapy in the current state of clinical trials are shown in Table 4.

Despite the success of preclinical research in identifying the roles of specific HDAC members in cancer and other physiological pathogenesis, there has been a gap in developing selective HDACi. The development in this area still remains in its nascent phase and mostly limited to preclinical research. Among the 18 HDAC members identified so far, only a handful of HDAC members have gained success in developing selective inhibitors by demonstrating strong preclinical antitumor effects. In the following concluding section, we will discuss examples of specific HDAC members with unmet needs to develop specific inhibitor as well as already developed specific HDAC inhibitors for preclinical uses. We have also incorporated their implications in the context of immune-related abnormality and/or specific malignancies. We strongly believe that these preclinical studies will pave successful avenues for novel and promising clinical trials in the future.

### 10.1. HDAC4

In addition to cancer, the role of HDAC4 has been extensively studied in the context of neurodegenerative diseases. Although HDAC4 is shuttled between the nucleus and cytoplasm, in brain cells it is exclusively located within the nucleus [161]. HDAC4 plays a significant role as a transcriptional repressor determining the in synaptic plasticity, which led to the investigation of HDAC4i for neuronal regulation under pathological conditions [162]. Several tumor types also feature an overexpression of HDAC4 as a prognostic marker of poor survival and aggressive tumor progression. Such overexpression has been shown in esophageal squamous cell carcinoma (ESCC) tissues and cell lines [163]. HDAC4 promotes the progression of colon cancer [164] and gastric cancer [165] through the repression of p21. HDAC4 regulates HIF1α protein acetylation and stability thereby promoting the hypoxia-related increase of glycolysis and resistance to docetaxel chemotherapy [166]. However, there is no selective HDAC4 inhibitor to date that necessitates the use of SAHA or TSA to target HDAC4 for both neurodegenerative disease and cancer [161]. Thus, the example of HDAC4 stands as an unmet need in the field, which could offer a lucrative option for further pharmaceutical development in the field of specific HDACi.

### 10.2. HDAC8

The phosphorylated form of HDAC8 is located in both the cytoplasm and nucleus of cancerous cells such as HeLa, whereas in the normal cells (HEK293) it is primarily found in the cytoplasm. The same study found HDAC8 to bind and deacetylate α-tubulin at the ac-lys40 residue. Moreover, overexpression of HDAC8 demonstrated a functional redundancy with HDAC6 [167]. A novel inhibitor of HDAC8 PCI-34051, which possesses >200-fold selectivity over the other HDAC isoforms, induced caspase-dependent apoptosis in T cell lines derived from lymphomas or leukemias. However, this effect was not observed in other hematopoietic or solid tumor lines. A potential mechanism for the induction of apoptosis could be the rapid intracellular calcium mobilization from the endoplasmic reticulum (ER) and subsequent release of cytochrome c from mitochondria [168]. Utilizing a PLCgamma1-defective cell line, HDAC8 was also associated with the activation of the PLCgamma1 pathway [168]. Outside the realm of cancer, HDAC8 has been associated with other pathogenic conditions, such as X-linked intellectual disability [169], and parasitic infections [170]. Despite their preclinical success in T cell malignancy, genetic and infectious diseases [171], HDAC8 inhibitors have not progressed to clinical trials yet.

### 10.3. HDAC11

Being the most recently identified HDAC member, HDAC11 is a zinc-dependent HDAC overexpressed in several carcinomas compared to the healthy tissues. As shown in colon (HCT-116), prostate (PC-3), breast (MCF7), and ovarian (SK-OV-3) cancer cell lines, depletion of HDAC11 to cause cell death and inhibition of metabolic activity whereas the normal cells appear to be immune to such effects [172]. In the context of metabolic regulation, HDAC11 has been directly associated with insulin sensitivity, glucose tolerance, hypercholesterolemia, and hepatosteatosis and liver damage since the depletion of HDAC11 attenuated these effects [173]. A selective inhibitor of HDAC11 quisinostat (QS) exhibited dose-dependent cytotoxicity on multiple myeloma cell lines. The compound showed a synergistic effect in combination with bortezomib and carfilzomib in using the RPMI-8226 and BTZ-resistant RPMI-8226-B25 cells. Genetic knockdown experiments showed the effect of HDAC11 on caspase 3 and, in turn, the cellular viability [174]. In the context of immune cells, HDAC11 was found to downregulate interleukin 10 (IL-10) expression in APCs that had significant implication for the inflammatory APCs and priming of naive T cells [175]. HDAC11 deficient FOXP3+ exhibits enhanced suppressive function along with a significant increase in the expression of FOXP3 and TGF-β. Selective HDAC11i JB3-22 reversed the inhibitory effect of HDAC11 on p300-induced FOXP3 acetylation at lysine 31 [176]. 

### 10.4. HDAC6

It occupies an interesting role in tumor immunology by exerting effects on both tumor and APC sides in the tumor microenvironment. On the one hand, HDAC6 forms a complex with STAT3 modulating its phosphorylation status in the macrophage and DC, which facilitates the production of IL10. Pharmacological inhibition of HDAC6, therefore, offered an avenue for bypassing the anti-inflammatory or tolerogenic mechanisms posed by tumors [62]. On the other hand, HDAC6 is necessary to maintain the tumor cells’ ability for anchorage-independent growth [177], cell cycle regulation [178], cell shape maintenance and cargo transport [179,180], and cellular stress response by regulating Hsp90 [181]. In more recent work on melanoma, HDAC6 was also found to be regulating the expression of PD-L1 in a STAT3-dependent manner now established in multiple tumor types. While primarily observed in melanoma [182], this relationship is now also established in osteosarcoma [183] and chronic lymphocytic leukemia (CLL) [184]. This observation opened a novel avenue of combining HDAC6 inhibition with the immunotherapeutic modality of PD1/PDL1 pathway. While the genetic knockdown and pharmacological inhibition by the HDAC6i nexturastat A markedly reduced melanoma tumor growth, the combination further resulted in (1) additive effect in reducing tumor growth compared to the individual effect of each agent, (2) higher infiltration of activated cytotoxic T cells, and (3) reduction in the anti-inflammatory properties of TAMs [10]. The combination also diminished the excessive production of intratumoral IFNγ, which is one of the byproducts of anti-PD1 antibody and is reported to enhance PD-L1 expression in the TME [185]. On the other hand, tubastatin A, another HDAC6i, has been shown to exert neuroprotective effect against oxidative stress via the acetylation of the redox regulatory protein peroxiredoxin 1 (Prx1) [186]. Taking all these observations into the account, the development and clinical application of HDAC6i may offer a viable option to complement the T cell-dependent immunotherapeutic modality in cancer as well as other inflammation-dependent pathological conditions.

Below is a compilation and comparison of pan HDACi with selective HDACi functions, as single or immunotherapeutic combinations that can be exploited in future cancer therapy (Table 5). We chose to use HDAC6 inhibition as a model due to its multifaceted role in the APC, T cells, and tumoral compartments. 

## 11. Final Remarks

It is evident that epigenetic and immunological alterations can lead to disease and malignancy. While immunotherapy has changed the approach to anticancer therapy, the toxicity profile (arising from cytokine storm, decrease in blood cell count, cardiac toxicity including ventricular arrhythmia, fatigue, and vomiting [106,187], and mechanisms of developing resistance, such as accrual of mutations [188], high levels of thioredoxin, or gain of pro-survival molecules such as BCL-2 [188]) cannot be ignored. As seen in hematological and solid malignancies, HDACi can revert epigenetic changes in a targeted manner. Copious research on HDAC molecules has allowed for HDACis to become realistic therapeutic agents for a wide array of diseases including cancer, autoimmune disease, and neurological damage, all the while retaining a narrower toxicity profile compared to other anti-cancer modalities, such as, chemotherapy. Their ability to alter various cellular pathways renders them ideal candidates to act as both single agents and as combination therapies with immunological modulators and chemo-immunotherapy, setting the stage for alternatives to chemotherapy. More work is needed to better understand the in vivo effect of selective HDACis. The pharmacokinetics of HDACis, like other biological agents, can be measured by their potency (measure of drug activity expressed in terms of the amount required to produce an effect of given intensity; defined by the range between the minimum effective dose (MED) and the maximum tolerated dose (MTD) [189], and their therapeutic indices (quantitative measurement of the relative safety of a drug; the ratio of the dose that elicits a lethal response in 50 percent of treated individuals (LD50) divided by the dose that elicits a therapeutic response in 50 percent of the treated individuals (TD50)) [190]. Unfortunately, data from the preclinical studies often lack information on these specific parameters. Nonetheless, data from such studies are indicatives of the efficacy and lower toxicity profiles that are brought about by these new agents onto animal models. With the advancement of biochemical technology, gathering more information through preclinical studies will be an attainable goal and will help us in optimizing clinical care using these new epigenetic modulators. 

## Figures and Tables

**Table 1:** Specificity and application of pan and selective HDACis.

	Class I				Class IIa				Class IIb		Class IV		Clinicaltrials.gov
Inhibitor Name	HDAC1	HDAC2	HDAC3	HDAC8	HDAC4	HDAC5	HDAC7	HDAC9	HDAC6	HDAC10	HDAC11		(02/22/2019)
**Vorinostat (SAHA, MK0683)**	++++	++++	++++	++++	++++	++++	++++	++++	++++	++++	++++	**Merck (FDA)**	251
**Panobinostat (LBH589)**	++++	++++	++++	++++	++++	++++	++++	++++	++++	++++	++++	**Novartis (FDA)**	133
**Trichostatin A (TSA)**	++++	++++	++++	++++	++++	++++	++++	++++	++++	++++	++++		15
**Belinostat (PXD101)**	++++	++++	++++	++++	++++	++++	++++	++++	++++	++++	++++	**TopoTarget (FDA)**	44
**LAQ824 (Dacinostat)**	++++	++++	++++	++++	++++	++++	++++	++++	++++	++++	++++	**Novartis**	-
**M344**	++++	++++	++++	++++	++++	++++	++++	++++	++++	++++	++++		-
**AR-42**	++++	++++	++++	++++	++++	++++	++++	++++	++++	++++	++++	**Arno Theraputics**	5
**Quisinostat (JNJ-26481585) 2HCl**	++++	++++	++++	++++	++++	++++	++	+++	++	++++	++++		6
**CUDC-907**	++++	++++	++++	++	++	+	++	++	+++	++++	++++		6
**Pracinostat (SB939)**	+++	++	+++	++	+++	+++	++	++	+	+++	++	**MEI Pharma (FDA)**	12
**CUDC-101**	++++	+++	++++	++	+++	+++	++	++	++++	+++		**Curis**	4
**Ricolinostat (ACY-1215)**	++	+++	+++	++	+	+	+		++++			**Celgene/Acetylon**	9
**PCI-24781 (Abexinostat)**	++++	+++	++++	++					+++	+++		**Pharmacyclics**	9
**HPOB**	+	+	+	+					+++	+			1
**MC1568**	++	++	++	++									-
**Mocetinostat (MGCD0103)**	++	++	+								+	**Mirati (FDA)**	22
**TMP269**					++	++	+++	+++					-
**PCI-34051**	+			++++					+	+			-
**Droxinostat**			+	+					+				-
**Resminostat**	+++		+++						++			**4SC**	5
**BRD73954**		+		++					+++				-
**BG45**	+	+	++										-
**4SC-202**	+	+	+									**4SC**	3
**CI994 (Tacedinaline)**	+	+	+										3
**LMK-235**					+++	++++							-
**Romidepsin (FK228, Depsipeptide)**	+++	+++										**Celgene**	88
**RG2833 (RGFP109)**	+++		++++									**Replign**	-
**Entinostat (MS-275)**	++		+									**Syndax**	60
**CAY10603**	++								++++				-
**Tubacin**									++++				-
**RGFP966**			++										-
**Tubastatin A**									+++				-
**Nexturastat A**									++++				-
**SS-2-08**									++++				-

**Table 2:** Examples of selected benefits of combination therapy.

Advantage	Example	References
Overcome resistance	Resistance to specific inhibitors can be overcome by combining with checkpoint inhibitor. 1. BRAF mutant melanoma developing resistance to BRAF inhibitor, achieved prolonged survival with Nivolumab. 2. Nivolumab + veliparib + Platinum Doublet Chemotherapy (Metastatic NSCLC)	NCT01721746 (Checkmate 037) NCT02944396
Increase efficacy and prolong survival	Targeting non-overlapping pathways to restore T cell function: nivolumab and ipilimumab combination; CTLA-4 blockade diminishes the CTLA-4 upregulation which may partially contribute to the resistance to PD-1 blockade.	[68,69] Currently 284 studies with dual checkpoint inhibitors registered in clinicaltrials.gov
Increase the span of target disease types	Combining checkpoint inhibitors with specific targeted therapy such as, Anti-PDL1 + anti-VEGF (for RCC), Anti-PD1 + BRAF inhibitor (for melanoma)	NCT02420821 (IMmotion151) NCT03625141
Engage different arms of the immune system	For locally advanced, recurrent, or metastatic incurable malignancies which have failed standard therapy due to insufficiency, treatment resistance, intolerance. E.g., anti PDL1 + IDO inhibitor in recurrent metastatic solid tumor, combining vaccine Viagenpumatucel-L with ICI Nivolumab for NSCLC	NCT02471846 NCT02439450

**Table 3:** Compilation of recent clinical trials with combination of pan-HDACi and immunotherapeutic/chemo-immune agents.

Combination	Cancer Type	Phase	Trial Identifier	Trials Status	Agency
Vorinostat + Pembrolizumab	Recurrent unresectable/metastatic HNSCC and SGC	I/II	NCT02538510	Active, not recruiting	U Washington + NCI
Vorinostat + Pembrolizumab	Lung Cancer, Stage IV NSCLC	I/II	NCT02638090	Recruiting	H. Lee Moffitt Cancer Center and Research Institute + Merck Sharp & Dohme Corp.
Vorinostat + Pembrolizumab	Advanced renal or urothelial cell	I/Ib	NCT02619253	Recruiting	Roberto Pili, Indiana University School of Medicine
Vorinostat + Pembrolizumab + Tamoxifen	Hormone resistant BC	II	NCT02395627	Active, not recruiting	Pamela Munster, University of California
Pembrolizumab + Vorinostat + Temozolomide	Glioblastoma	I	NCT03426891	Recruiting	H. Lee Moffitt Cancer Center + Merck Sharp & Dohme Corp.
Vorinostat + Exemestane	ER+ breast cancer	II	NCT00676663	Completed	Syndax pharmaceuticals
Vorinostat + Bortezomib	Recurrent Mantle Cell Lymphoma or Recurrent and/or Refractory Diffuse Large B-Cell Lymphoma	II	NCT00703664	Completed	NCI
Vorinostat, Paclitaxel, and Radiation Therapy	Stage IIIA/B unresectable non-small cell lung cancer who can’t tolerate cisplatin	I/II	NCT00662311	Terminated	U Washington + NCI
Vorinostat + Bortezomib	Multiple myeloma	III	NCT00773747	Completed	Merck Sharp & Dohme Corp.
Vorinostat + Gemtuzumab ozogamicin	Acute myeloid leukemia (older patients without prior treatment)	II	NCT00673153	Terminated	Fred Hutchinson Cancer Research Center + NCI
Vorinostat + Dexamethasone + Bortezomib	Relapsed or Refractory Multiple Myeloma	II	NCT00773838	Completed	Merck Sharp & Dohme Corp.
Vorinostat + Olaparib	Relapsed/Refractory and/or Metastatic Breast Cancer	I	NCT03742245	Not yet recruiting	Jenny C. Chang + Aztrazeneca
Vorinostat + gefitinib	Resistance by BIM Polymorphysim in EGFR Mutant Lung Cancer	I	NCT02151721	Active, not recruiting	Kanazawa University
Entinostat + Exemestane	Postmenopausal Women Patients with Locally Recurrent or Metastatic Breast Cancer	I	NCT02833155	Recruiting	EddingPharm Oncology
Entinostat + Atezolizumab	Phase 1b TNBC	I/II	NCT02708680	Active, not recruiting	Syndax Pharmaceuticals + Roche
Entinostat + Avelumab	Advanced Epithelial Ovarian Cancer	Ib/2	NCT02915523	Active, not recruiting	Syndax Pharmaceuticals +Merck KGaA, Darmstadt, Germany Pfizer
Entinostat + Nivolumab	Children and Adolescents with High-risk Refractory Malignancies	I/II	NCT03838042	Not yet recruiting	University Hospital Heidelberg + Geramn Cancer Research Center
Entinostat + Pembrolizumab	Non-Small Cell Lung Cancer, Melanoma, Mismatch Repair-Proficient Colorectal Cancer	I/II	NCT02437136	Not yet recruiting	Syndax Pharmaceuticals + Merck Sharp & Dohme Corp.
Entinostat, Lapatinib Ditosylate and Trastuzumab	HER2/Neu Positive Invasive Breast Carcinoma Recurrent Breast Carcinoma Stage IV Breast Cancer AJCC v6 and v7	I	NCT01434303	Active, not recruiting	NCI
Panobinostat + Trastuzumab	HER2 Positive Metastatic Breast Cancer, pretreated with Transtuzumab	I/II	NCT00567879	Terminated	Novartis
Panobinostat Given in Combination with Trastuzumab and Paclitaxel	HER-2 Positive Breast Cancer	I	NCT00788931	Completed	Novartis
Pembrolizumab + Guadecitabine + Mocetinostat	Advanced lung cancer	I	NCT03220477	Recruiting	Memorial Sloan Kettering Cancer Center + Merck Sharp & Dohme Corp. Astex Pharmaceuticals. Mirati Therapeutics Inc. Stand Up To Cancer Van Andel Research Institute
Glesatinib + Sitravatinib + Mocetinostat + Nivolumab	Carcinoma, Non-Small-Cell Lung	II	NCT02954991	Recruiting	Mirati pharmaceuticals
Romidepsin + Nivolumab	Metastatic TNBC	I/II	NCT02393794	Recruiting	Priyanka Sharma, U of Kansas + Celgene Corporation Bristol-Myers Squibb

**Table 4:** Examples of clinical trials with selective HDACis in combination with immunotherapeutic/chemo-immune agents.

Combination	Cancer Type	Phase	Trial Identifier	Trials Status	Agency
MPT0E028	Advanced Solid Malignancies Without Standard Treatment	I	NCT02350868	Recruiting	Taipei medical University
Chidamide Maintenance After Autologous Hematopoietic Stem Cell Transplantation	Relapsed, Refractory or High-risk Lymphoma	II	NCT03611231	Not yet recruiting	Peking University + Hebei Medical University Fourth Hospital + Peking University International Hospital
Chidamide Combined with Clad/Gem/Bu With AutoSCT	High Risk Hodgkin & Non-Hodgkin Lymphoma	II	NCT03602131	Not yet recruiting	Sichuan University
Ricolinostat in Combination with Pomalidomide and Dexamethasone	Relapsed or Relapsed-and-Refractory Multiple Myeloma	I	NCT02189343	Active, not recruiting	Celgene
Ricolinostat Alone and in Combination with Bortezomib and Dexamethasone	Multiple myeloma	I/II	NCT01323751	Completed	Celgene

**Table 5:** Comparison of pan-HDACi and class 1 HDACi to a selective HDACi in the context of immune functions.

	pan HDACi	Class I HDACi	HDAC6i
**Tumor**	↑ MHC and Antigen Presentation [7,58,65] Cellular Toxicity [144,145,146] ↑ Immunosupressive pathways [29,33,151] ↑ Tumor Associated Antigens [152]	↑ MHC and Antigen Presentation [7] ↑ Immunosupressive Pathways [147,148,149]	↓ Immunosuppressive pathways [138,139,141] ↑ MHC and Antigen Presentation [150] ↑ Tumor Associated Antigens [150]
**APC**	Cellular Toxicity [153] ↓ Inflammatory mediators [156] ↑ MHC and Antigen Presentation [7,58,65] Modulate APC Function [62] ↑ Immunosupressive pathways [163,164]	↑ Immunosupressive pathways [148,149,154,155] ↑ MHC and Antigen Presentation [157,158] Modulates APC Function [160,161,162]	↓ Immunosupressive pathways [138,139,141] ↑ MHC and Antigen Presentation [159]
**T Cell (effectors)**	↓ Inflammatory mediators [33,165] ↑ T cell functionality [85,167] ↑ Chemokine production [85]	Cellular Toxicity [166] ↓ Proliferation [158,166,168]	Minimal Changes [141]
**Treg**	↓ Proliferation [151] ↓ Suppressive function [151]	↓ Proliferation [5] ↓ Suppresive function [155]	↑Suppresive function [169,170]
**MDSC**	Cellular Toxicity [164]	↓ Suppresive Function [154]	ND
